# Literary Notices

**Published:** 1885-02

**Authors:** 


LITERARY NOTICES.

Owing to the illness of Dr. Albert H. Smith, of Philadelphia, Dr. E. C. Dudley, of Chicago, has been invited to contribute an article upon "Displacements" to Pepper's "System of Practical Medicine, by American Authors."

The editor is to be congratulated upon this accession to the ranks of eminent American practitioners, who are already enrolled.

Messrs. P. Blackiston, Son & Co., of Philadelphia, are about to publish a very important medical book, entitled "Clinical Studies of Diseases of the Eye, including the Conjunctiva, Cornea, Sclerotic, Iris, and Ciliary Body," by Dr. Ferd. Ritter von Arlt, *Professor der Augenheilkunde in Wien*, translated by Dr. Lyman Ware, Surgeon to the Illinois Charitable Eye and Ear Infirmary; Opthalmic Surgeon to Presbyterian Hospital and Protestant Orphan Asylum, Chicago.

